# The Role of Balanced Time Perspective in Migraine Pain Relief

**DOI:** 10.1192/j.eurpsy.2026.10930

**Published:** 2026-07-31

**Authors:** E. Plucińska, M. Sobol, P. Wrona, M. Boczarska-Jedynak, J. Piotrowska, P. Bąbel, J. Howick, N. Maleki

**Affiliations:** 1 Faculty of Psychology, University of Warsaw, Warsaw; 2 Department of Psychiatry, PZOL, Miedzybrodzie Bialskie; 3 Department of Neurology, Jagiellonian University Medical College, Krakow; 4 Health Institute, Oswiecim; 5 SMOK Medical Center, Warsaw; 6 Jagiellonian University, Krakow, Poland; 7 University of Leicester, Leicester, United Kingdom; 8 Massachusetts General Hospital, Boston, United States

## Abstract

**Introduction:**

Migraines are a complex neurobiological disorder, ranking as the third most common disease worldwide. Among the most frequently reported triggers of migraines is stress, which has been shown to prolong headache duration and increase the risk of chronification (Seng et al. Lancet Neurol. 2022; 21(10) 911-921). Time perspective is defined as an individual’s cognitive and emotional orientation toward time, encompassing past, present, and future dimensions. Balanced Time Perspective (BTP) is associated with cognitive and behavioral flexibility, enabling individuals to adapt their temporal focus according to situational demands. Within this framework, psychological flexibility appears to be a critical protective factor that can prevent migraine attacks. We investigated BTP as a moderator of the relationship between perceived stress and migraine.

**Objectives:**

The aim of this study was to examine the association of perceived stress and balanced time perspective (BTP) with migraine frequency, severity, and migraine-related disability. Accordingly, it was hypothesized that BTP would be inversely related to the frequency of migraine attacks, the severity of migraine-related pain, and the level of migraine-related disability.

**Methods:**

This study was conducted between November 2024 and May 2025 in Poland. A total of 91 adult participants who were diagnosed with migraines. Participants were eligible for inclusion if they were 18 years or older and had received a migraine diagnosis. Participants completed a set of questionnaires, including the Zimbardo Time Perspective Inventory, Carpe Diem Scale, Dark Future Scale, Migraine Disability Assessment Score questionnaire, and Perceived Stress Scale. They also underwent migraine diaries for 4 weeks.

**Results:**

The results of the moderation analysis indicated that BTP was a moderator of the relationship between perceived stress and migraine related disability measured by the MIDAS, as well as migraine frequency measured by the MIDAS. No associations were found between BTP and migraine indicators recorded in the migraine diaries. These findings may be interpreted within the framework of pain memory, where frequent activation of pain-related memories can intensify current pain.

**Conclusions:**

The findings of the present study provide further empirical evidence for the influence of psychological factors in the progression of migraines. In particular, the results indicate that BTP may serve as a psychological buffer, reducing the likelihood of migraine episodes during periods of elevated stress. These findings also suggest that psychological interventions aimed at enhancing BTP could represent a promising approach to mitigating migraine symptoms.

**Disclosure of Interest:**

E. Plucińska Grant / Research support from: Preparation of this manuscript was supported by the grant 2023/49/B/HS6/03227, Opus 25 from the National Science Centre (Poland)., M. Sobol Grant / Research support from: Preparation of this manuscript was supported by the grant 2023/49/B/HS6/03227, Opus 25 from the National Science Centre (Poland)., P. Wrona: None Declared, M. Boczarska- Jedynak: None Declared, J. Piotrowska: None Declared, P. Bąbel: None Declared, J. Howick: None Declared, N. Maleki: None Declared

